# *Culex quinquefasciatus* from Rio de Janeiro Is Not Competent to Transmit the Local Zika Virus

**DOI:** 10.1371/journal.pntd.0004993

**Published:** 2016-09-06

**Authors:** Rosilainy Surubi Fernandes, Stéphanie Silva Campos, Anielly Ferreira-de-Brito, Rafaella Moraes de Miranda, Keli Antunes Barbosa da Silva, Marcia Gonçalves de Castro, Lidiane M. S. Raphael, Patrícia Brasil, Anna-Bella Failloux, Myrna C. Bonaldo, Ricardo Lourenço-de-Oliveira

**Affiliations:** 1 Laboratório de Mosquitos Transmissores de Hematozoários, Instituto Oswaldo Cruz, Rio de Janeiro, Brazil; 2 Laboratório de Biologia Molecular de Flavivírus, Instituto Oswaldo Cruz, Fiocruz, Rio de Janeiro, Brazil; 3 Laboratório de Pesquisa Clínica em Doenças Febris Agudas, Instituto Nacional de Infectologia, Rio de Janeiro, Brazil; 4 Institut Pasteur, Arboviruses and Insect Vectors, Paris, France; The Connecticut Agricultural Experiment Station, UNITED STATES

## Abstract

**Background:**

The Americas have suffered a dramatic epidemic of Zika since May in 2015, when Zika virus (ZIKV) was first detected in Brazil. Mosquitoes belonging to subgenus *Stegomyia* of *Aedes*, particularly *Aedes aegypti*, are considered the primary vectors of ZIKV. However, the rapid spread of the virus across the continent raised several concerns about the transmission dynamics, especially about potential mosquito vectors. The purpose of this work was to assess the vector competence of the house mosquito *Culex quinquefasciatus* from an epidemic Zika area, Rio de Janeiro, Brazil, for local circulating ZIKV isolates.

**Methodology/Principal Findings:**

*Culex quinquefasciatus* and *Ae*. *aegypti* (positive control of ZIKV infection) from Rio de Janeiro were orally exposed to two ZIKV strains isolated from human cases from Rio de Janeiro (Rio-U1 and Rio-S1). Fully engorged mosquitoes were held in incubators at 26 ± 1°C, 12 h:12 h light:dark cycle and 70 ± 10% humidity. For each combination mosquito population—ZIKV strain, 30 specimens were examined for infection, dissemination and transmission rates, at 7, 14 and 21 days after virus exposure by analyzing body (thorax plus abdomen), head and saliva respectively. Infection rates were minimal to completely absent in all *Cx*. *quinquefasciatus*-virus combinations and were significantly high for *Ae*. *aegypti*. Moreover, dissemination and transmission were not detected in any *Cx*. *quinquefasciatus* mosquitoes whatever the incubation period and the ZIKV isolate. In contrast, *Ae*. *aegypti* ensured high viral dissemination and moderate to very high transmission.

**Conclusions/Significance:**

The southern house mosquito *Cx*. *quinquefasciatus* from Rio de Janeiro was not competent to transmit local strains of ZIKV. Thus, there is no experimental evidence that *Cx*. *quinquefasciatus* likely plays a role in the ZIKV transmission. Consequently, at least in Rio, mosquito control to reduce ZIKV transmission should remain focused on *Ae*. *aegypti*.

## Author Summary

The pandemic Zika epidemic has affected nearly all American countries. The etiological agent is a mosquito-borne-virus originated from Africa that spread to Asia and more recently, to the Pacific region and the Americas. We experimentally demonstrated that the common house nightly biting mosquito *Culex quinquefasciatus* from Rio de Janeiro was not susceptible to locally circulating Zika virus (ZIKV) strains. Dissemination was not observed in *Cx*. *quinquefasciatus* regardless of the ZIKV isolate used and the incubation period after the ingestion of an infected blood meal. No infectious ZIKV particle was detected in the saliva of the four *Cx*. *quinquefasciatus* populations examined until 3 weeks after virus exposure. In contrast, we confirmed that local *Aedes aegypti* mosquitoes can be infected, disseminate ZIKV at significantly high rates, and assured moderate to very high viral transmission after day 14 of virus exposure. We concluded that *Cx*. *quinquefasciatus* is not competent to transmit local ZIKV. Our results support that mosquito control should focus on *Ae*. *aegypti* to reduce Zika transmission.

## Introduction

A Zika virus (ZIKV) epidemic has rapidly spread throughout tropical and subtropical zones of the American continent since early 2015 [1]. Brazil was likely the starting point of the Zika pandemic in the Americas [2, 3]. The Zika virus pandemic has spread to North America too. By July 2016, 45 American countries or territories have already reported active ZIKV transmission (http://www.cdc.gov/zika/geo/active-countries.html).

ZIKV is a positive-sense, single-stranded RNA mosquito-borne-virus of 10,807 nucleotides belonging to family *Flaviviridae*, genus *Flavivirus*. It is composed of three major lineages: East African, West African, and Asian [4]. ZIKV was first isolated from a sentinel rhesus monkey in the Zika forest in Uganda in 1947 [5]. The second ZIKV isolations were obtained from 20 pools of the forest canopy feeder mosquito *Aedes (Stegomyia) africanus* captured in the same area [6].

Almost 70 years have passed and little is known about natural ZIKV vectors. *Aedes* mosquitoes are considered the primary vectors of ZIKV in Africa with reported viral isolations from several species, especially from *Ae*. *africanus* [1, 7–10]. ZIKV was also isolated from several other mosquito species belonging to genus *Aedes* (subgenera *Stegomyia* and *Diceromyia*), *Mansonia* and *Culex*, and horse flies from the wild in Uganda [8]. More recently, natural infections screened by molecular methods in sylvatic African mosquitoes were again predominantly found in *Aedes* belonging to subgenus *Stegomyia*, but also in other species of *Aedes*, *Mansonia*, *Culex*, *Anopheles* [9, 10]. Nevertheless, ZIKV transmission in the wild has remained poorly understood. Only two sylvatic species (*Ae*. *vittatus* and *Ae*. *luteocephalus*) proved to be able to transmit ZIKV in laboratory assays [11].

The domestic mosquito *Ae*. *(Stegomyia) aegypti* was early shown to be competent to experimentally transmit ZIKV [12]. Due to its high anthropophilic and domestic behaviors and virus detection in field caught specimens [13, 14], this mosquito has been incriminated as the urban and periurban vector in Africa and Asia [1,15].

ZIKV has only recently emerged outside of its natural distribution in Africa and Asia, and has caused a series of epidemics in urban and periurban sites on Pacific islands [16–20] before reaching the Americas, probably in 2013 [21]. The spreading virus belonged to the Asian genotype [21]. Despite multiple efforts, mosquito vectors involved in the ZIKV outbreaks across the Pacific Ocean in 2007–2015 were not identified. *Ae*. *aegypti* and other local members of subgenus *Stegomyia* (*Ae*. *hensilli* and *Ae*. *polynesiensis*) were thought to be potential vectors [16, 22, 23]. *Ae*. (*Stegomyia*) *albopictus* was found naturally infected with ZIKV in urban sites in Gabon in 2007 [24] and Mexico (http://www.paho.org/hq/index.php?option=com_docman&task=doc_view&Itemid=270&gid=34243&lang=en). Additionally, *Ae*. *aegypti* from Singapore were competent to transmit the African ZIKV genotype in the laboratory [25]. Thereafter, *Ae*. *albopictus* has been considered a potential vector of ZIKV throughout its geographical range, concomitantly or not with *Ae*. *aegypti* [1, 24, 26, 27].

With the arrival of the ZIKV Asian genotype in the Americas, the global number of suspected and confirmed ZIKV cases reached levels never seen previously [28, 29]. Besides, the rapid geographical spread, the increased incidence of severe congenital troubles, such as microcephaly, and Guillain-Barré syndrome associated with ZIKV in Brazil led the World Health Organization to declare the ZIKV epidemic a Public Health Emergency of International Concern [1, 30]. ZIKV proved to have a high potential for geographic expansion in regions wherever *Ae*. *aegypti* mosquitoes are present, concomitantly with Dengue viruses 1–4 and Chikungunya virus prone areas of transmission, as it has occurred in Brazil and other American tropical and subtropical countries [29, http://www.cdc.gov/zika/geo/active-countries.html]. American *Ae*. *aegypti* and *Ae*. *albopictus* populations showed to be competent to transmit the ZIKV belonging to the circulating genotype, but displayed heterogeneous infection, dissemination and transmission rates in laboratory assays [26]. However, *Ae*. *aegypti* and *Ae*. *albopictus* populations from Brazil and USA exhibited low transmission efficiency to ZIKV [26], which appeared inconsistent with the rapid Zika spread throughout the Americas. Two main hypotheses might explain this scenario: (1) The large number of humans susceptible to ZIKV combined with high densities of anthropophilic *Aedes* mosquitoes compensate their relatively low vector competence to ZIKV [26]. (2) Although the recent ZIKV pandemic has occurred only in *Stegomyia-*infested zones and *Ae*. *aegypti* has been suggested to be the main vector, other anthropophilic, domestic and usually abundant mosquitoes such as *Culex* species could contribute to ZIKV transmission [1, 31]. For example, *Culex* species belonging to the Pipiens Assemblage [32], such as *Cx*. *quinquefasciatus*, were likely candidate due their high human-biting frequency and distribution in urban epidemic centers (http://www.reuters.com/article/us-health-zika-brazil-idUSKCN0W52AW). There is no information whether *Cx*. *quinquefasciatus* can transmit the virus or the potential role of this mosquito in the ZIKV transmission in nature. We herein comparatively assess the vector competence of *Cx*. *quinquefasciatus* and *Ae*. *aegypti* populations from Rio de Janeiro for two local ZIKV isolates.

## Materials and Methods

### Mosquitoes

*Cx*. *quinquefasciatus* populations tested in this study were collected from four districts of Rio de Janeiro: Manguinhos (MAN, 22°52’20”S 43°14’46”W), Triagem (TRI, 22°53’56”S 43°14’44”W) Copacabana (COP, 22°58’8.3”S 43°11’21”W) and Jacarepaguá (JAC, 22°57’42”S 43°24’11”W). For comparison, we used two populations of *Ae*. *aegypti* from Rio de Janeiro, Brazil: Urca (URC, 22°56’45”S 43°09’43”W) and Paquetá (PAQ, 22°45’44”S 43°06’26”). The mosquitoes were concurrently collected as larvae or eggs using ovitraps from January to March 2016 to initiate laboratory colonies. Each colony was started with at least 200 field-collected individuals from more than five distinct collecting sites and traps. Field collected larvae and eggs were hatched and reared in insectaries (26 ± 1°C; 70 ± 10% RH; 12 h:12 h light:dark cycle). Larvae were reared in pans (~100 larvae/pan measuring 30 x 21 x 6 cm) containing 1 liter of dechlorinated tap water supplemented with yeast tablets. Adults were kept under the same insectary controlled conditions described above, and supplied with a 10% sucrose solution. All experimental oral infections were performed with mosquitoes of the F1 generation, except for TRI (laboratory colony) and PAQ (F2).

### Viral strains

Mosquitoes were challenged with two ZIKV strains of the Asian genotype, named Rio-U1 and Rio-S1, respectively isolated from urine and saliva of two patients in January 2016, living in distinct districts in Rio de Janeiro [33]. The viral samples were isolated, kept anonymized and provided by Bonaldo et al. [33], whose the institutional review board at Fundação Oswaldo Cruz has previously approved their study protocol. Viral stocks were obtained after two passages of the isolates onto Vero cells maintained with Earle’s 199 medium supplemented with 5% fetal bovine serum (FBS), under an atmosphere containing 5% CO_2_, and incubated at 37°C. Viral titer in supernatants were estimated by plaque-forming unit (PFU) assays of serial dilutions on Vero cells maintained at 37°C for 7 days and expressed in PFU/mL. Samples were kept at -80°C until use. The comparison of genomic sequences of ZIKV strains Rio-U1 (KU926309) with Rio-S1 (KU92630) yielded 99.6% identity, displaying six amino acid variations in the viral proteins. Phylogenetic analysis showed 99.7% identity of Rio-U1 and Rio-S1 strains with ZIKV isolates from Guatemala and other Brazil regions, including a Zika-associated microcephaly case. They all cluster (bootstrap score = 97%) within the Asian genotype and share a common ancestor with the ZIKV strain that circulated in French Polynesia in November 2013 [33].

### Mosquito experimental assays

Five to seven day-old females were isolated in feeding boxes and starved for 24 h and 48 h for *Aedes* and *Culex* mosquitoes, respectively. All mosquitoes were exposed to the infectious blood-meal containing a final viral titer of 10^6^ PFU/mL which consists of a mixture of two parts of washed rabbit erythrocytes and one part of the viral suspension added with a phagostimulant (0.5 mM ATP). Females were fed through a pig-gut membrane covering the base of glass feeders containing the infectious blood-meal maintained at 37°C. Mosquito feeding was limited to 60 min. Only fully engorged females were incubated at 26°C constant temperature, 70 ± 10% RH and 12 h:12 h light:dark cycle, with daily access to 10% sucrose solution. When available, samples of 30 mosquitoes of each population were examined at 7, 14 and 21 days after virus exposure, hereinafter abbreviated as “dpi”.

Mosquitoes were individually processed as follows: abdomen and thorax (herein after referred to as body) were examined to estimate viral infection rate, head for dissemination and saliva for transmission. Each female was handled at a time, by using disposable and disinfected supplies to avoid contamination between individuals and between tissues of the same mosquito as previously described [34]. For the determination of viral infection and dissemination rates, each mosquito body and head were respectively ground in 500 μL and 250 μL of medium supplemented with 4% FBS, and centrifuged at 10,000 x *g* for 5 min at +4°C before titration. Body and head homogenates were serially diluted and inoculated onto monolayers of Vero cells in 96-well plates. After 1 h incubation of homogenates at 37° C, 150 μL of 2.4% CMC (carboxymethyl cellulose) in Earle’s 199 medium was added per well. After 7 days incubation at 37° C, cells were fixed with 10% formaldehyde, washed, and stained with 0.4% crystal violet. Presence of viral particles was assessed by detection of viral plaques. Additionally, body and head homogenates were individually submitted to specific ZIKV RNA detection and quantification through RT-qPCR, using the SuperScript III Platinum one-step RT-qPCR (Invitrogen) in QuantStudio 6 Flex Real-Time PCR System (Applied Biosystems). For each reaction, we used 600 nM forward primer (5’-CTTGGAGTGCTTGTGATT-3’, genome position 3451–3468), 600 nM reverse primer (5’-CTCCTCCAGTGTTCATTT-3’, genome position 3637–3620) and 800 nM probe (5’FAM- AGAAGAGAATGACCACAAAGATCA-3’TAMRA, genome position 3494–3517). The sequences of this primer set were provided by Isabelle Lepark-Goffart (French National Reference Centre for Arboviruses, IRBA, Marseille, France). The reverse transcription was performed at 45° C for 15 min. The qPCR conditions were 95° C for 2 minutes, followed by 40 amplification cycles of 95° C for 15 sec, 58° C for 5 sec and 60° C for 30 sec. For each run, numbers of ZIKV RNA copies were calculated by absolute quantitation using a standard curve, whose construction details are described elsewhere [33].

In order to assess the transmission rate (TR) and transmission efficiency (TE), mosquito saliva was collected in individual pipette tips containing 5 μL FBS and processed by PFU assays, as previously described [26]. Accordingly, mosquito saliva was inoculated onto Vero Cell monolayer in 6-well plates incubated 7 days at 37° C, under 3 mL with 2.4% CMC in Earle’s 199 medium overlay, and stained as described above. Viral titers of saliva were expressed as PFU/saliva.

Infection rate (IR) refers to the proportion of mosquitoes with infected body (abdomen and thorax) among tested mosquitoes. Disseminated infection rate (DIR) corresponds to the proportion of mosquitoes with infected head among tested mosquitoes (i.e.; abdomen/thorax positive). Transmission efficiency (TE) represents the proportion of mosquitoes with infectious saliva among the initial number of mosquitoes tested. Transmission rate (TR) represents the proportion of mosquitoes with infectious saliva among mosquitoes with disseminated infection.

### Statistical analysis

To compare the viral load, the Wilcoxon signed rank test was adopted to analyze pairwise comparison at 7, 14 and 21 dpi for each mosquito population and tested virus strain. Significant difference was established when p-values were lower than 0.05. Data analyses were conducted with PRISM 5.0 software (GraphPad Software, San Diego-CA, USA, 2007).

### Ethics statements

This study was approved by the Institutional Ethics Committee on Animal Use (CEUA-IOC license LW-34/14) at the Instituto Oswaldo Cruz. No specific permits were required for performing mosquito collection in the districts in Rio de Janeiro.

## Results

### *Culex quinquefasciatus* infrequently become infected with ZIKV

**Fig 1:**
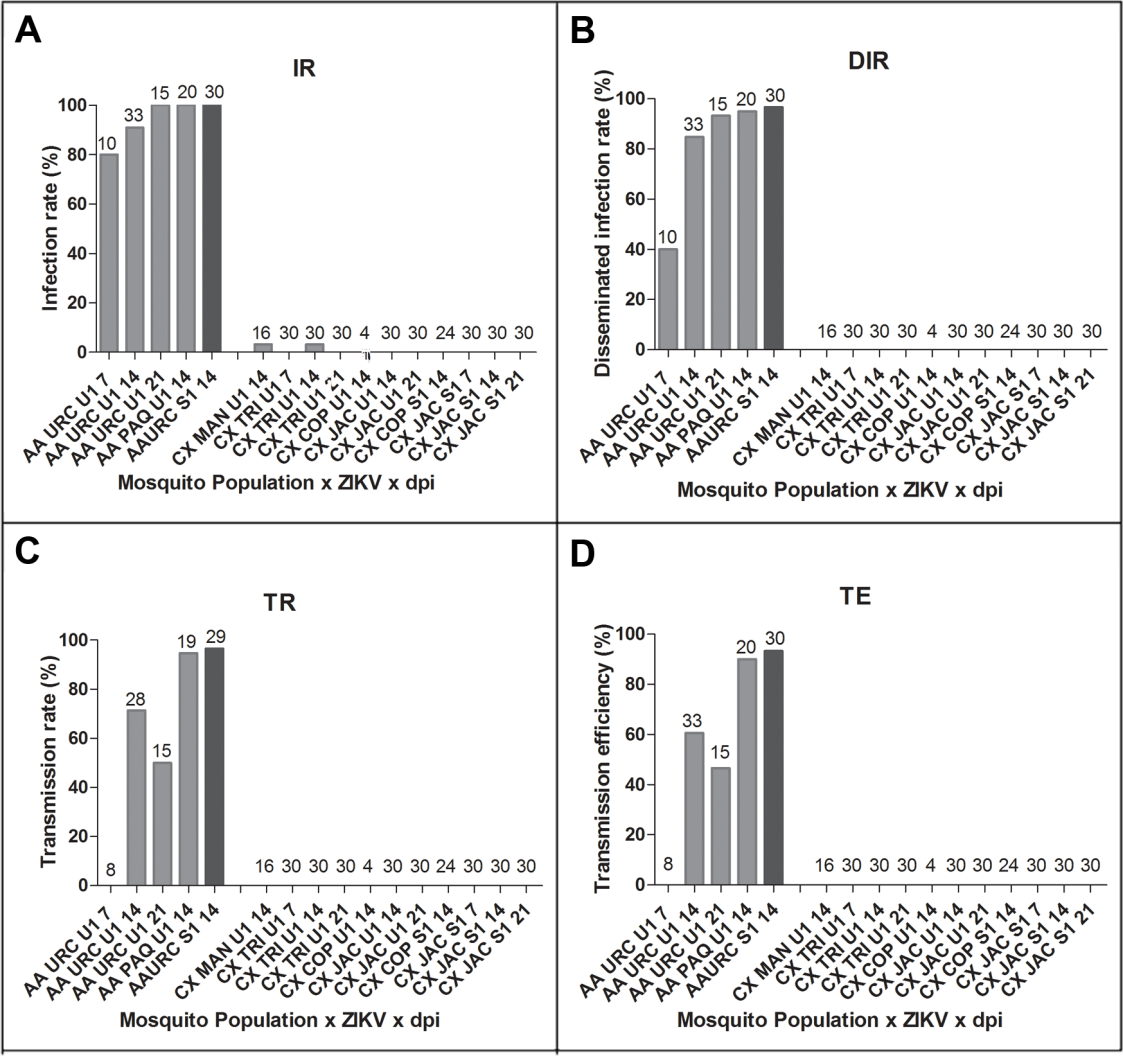
Viral infection (A), dissemination (B), transmission (C, D) at days 7, 14 and 21 after challenge of *Aedes aegypti* and *Culex quinquefasciatus* from Rio de Janeiro, Brazil, with two locally circulating ZIKV isolates (Rio-U1 and Rio-S1) provided at a titer of 10^6^ PFU/mL). Infection rate (IR) refers to the proportion of mosquitoes with infected body (abdomen and thorax) among tested mosquitoes. Disseminated infection rate (DIR) corresponds to the proportion of mosquitoes with infected head among tested mosquitoes (i.e.; abdomen/thorax positive). Transmission efficiency (TE) represents the proportion of mosquitoes with infectious saliva among the initial number of mosquitoes tested. Transmission rate (TR) represents the proportion of mosquitoes with infectious saliva among mosquitoes with disseminated infection. The number of individuals analyzed is given on top of bars.

**Fig 2:**
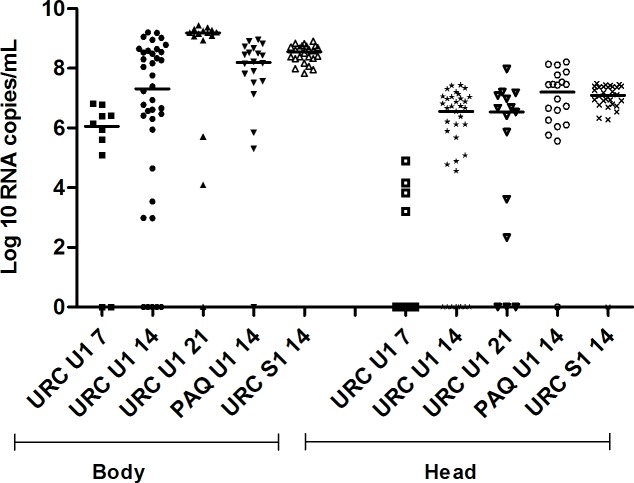
ZIKV load in bodies and heads of *Ae*. *aegypti* from Rio de Janeiro, Brazil, 7, 14 and 21 days after challenge with two locally circulating ZIKV isolates (Rio-U1 and Rio-S1) provided at a titer of 10^6^ PFU/mL). Viral RNA copies were determined by qPCR in mosquito homogenates. Viral loads with value 0 actually represents mosquitos with viral loads < 40 RNA copies/ml.

We comparatively evaluated the susceptibility to infection of *Cx*. *quinquefasciatus* and *Ae*. *aegypti* from Rio de Janeiro to two ZIKV strains locally isolated. Infection rates (IR) were negligible to null in *Cx*. *quinquefasciatus*, whereas they remained very high for *Ae*. *aegypti*, (Fig 1A). With few exceptions, the IRs were of 100% in the two tested *Ae*. *aegypti* populations (URC and PAQ) at 14 and 21 dpi, for both virus isolates. In addition, when examining *Ae*. *aegypti* from URC, 80% have already been infected by 7 dpi (Fig 1A). In contrast, none of the four *Cx*. *quinquefasciatus* populations was likely to become infected except for 1 of 30 TRI *Cx*. *quinquefasciatus* challenged with ZIKV Rio-U1, at 14 dpi (viral load: 1,814 RNA copies/ml; 7.0 PFU/ml) (Fig 1A). ZIKV RNA copies (1,453 RNA copies/ml) were detected in 1 of 16 MAN *Cx*. *quinquefasciatus* at 14 dpi challenged with the same ZIKV strain. However infective viral particles were not detected in the homogenate of this specimen in repeated PFU assays. Viral load estimated in bodies of *Ae*. *aegypti* tended to increase with incubation time (Fig 2), and the lowest values being detected at 7 dpi (median: 1.1 x 10^6^ RNA copies/ml, mean ± SE: 2.3 x 10^6^ ± 2.4 x 10^6^ RNA copies/ml) and the highest at 21 dpi (median: 1.5 x 10^9^ RNA copies/ml, mean ± SE: 1.3 x 10^9^ ± 8.3 x 10^8^ RNA copies/ml). Accordingly, viral load was significantly higher at 21 dpi than at 7 (p = 0.0098) and 14 dpi (p = 0.009). Viral loads at 14 dpi in bodies of *Ae*. *aegypti* from PAQ [IR: 100%, Fig 1; viral load: 1.6 x 10^8^ RNA copies/mL (median); 2.6 x 10^8^ ± 2.8 x 10^8^ RNA copies/mL (mean ± SE), Fig 2] were significantly higher than for URC [IR: 90.9%, Fig 1, viral load: 2.1 x 10^7^ RNA copies/mL (median); 2.6 x 10^8^ ± 4.3 x 10^8^ RNA copies/mL (mean ± SE), Fig 2] when challenged with the same ZIKV isolate (Rio-U1).

### The circulating ZIKV can promptly disseminate and efficiently be transmitted by *Ae*. *aegypti*, but not by *Cx*. *quinquefasciatus* from Rio

*Cx*. *quinquefasciatus* did not showed viral dissemination regardless of the incubation period whereas dissemination infection rates (DIR) were consistently high (~85–97%) in *Ae*. *aegypti* at 14 and 21 dpi irrespective the ZIKV strain (Fig 1B). Accordingly, transmission determined by detecting infective viral particles in mosquito saliva was not observed in any pair of *Cx*. *quinquefasciatus* population-ZIKV strain regardless the time point of examination (Fig 1C). In contrast, significantly high transmission rates (TR: 71.6–96.5%) and transmission efficiency (TE: 60.6–93.3%) were observed in local *Ae*. *aegypti* (PAC and URC) at 14 dpi (Fig 1C and 1D).

**Fig 3:**
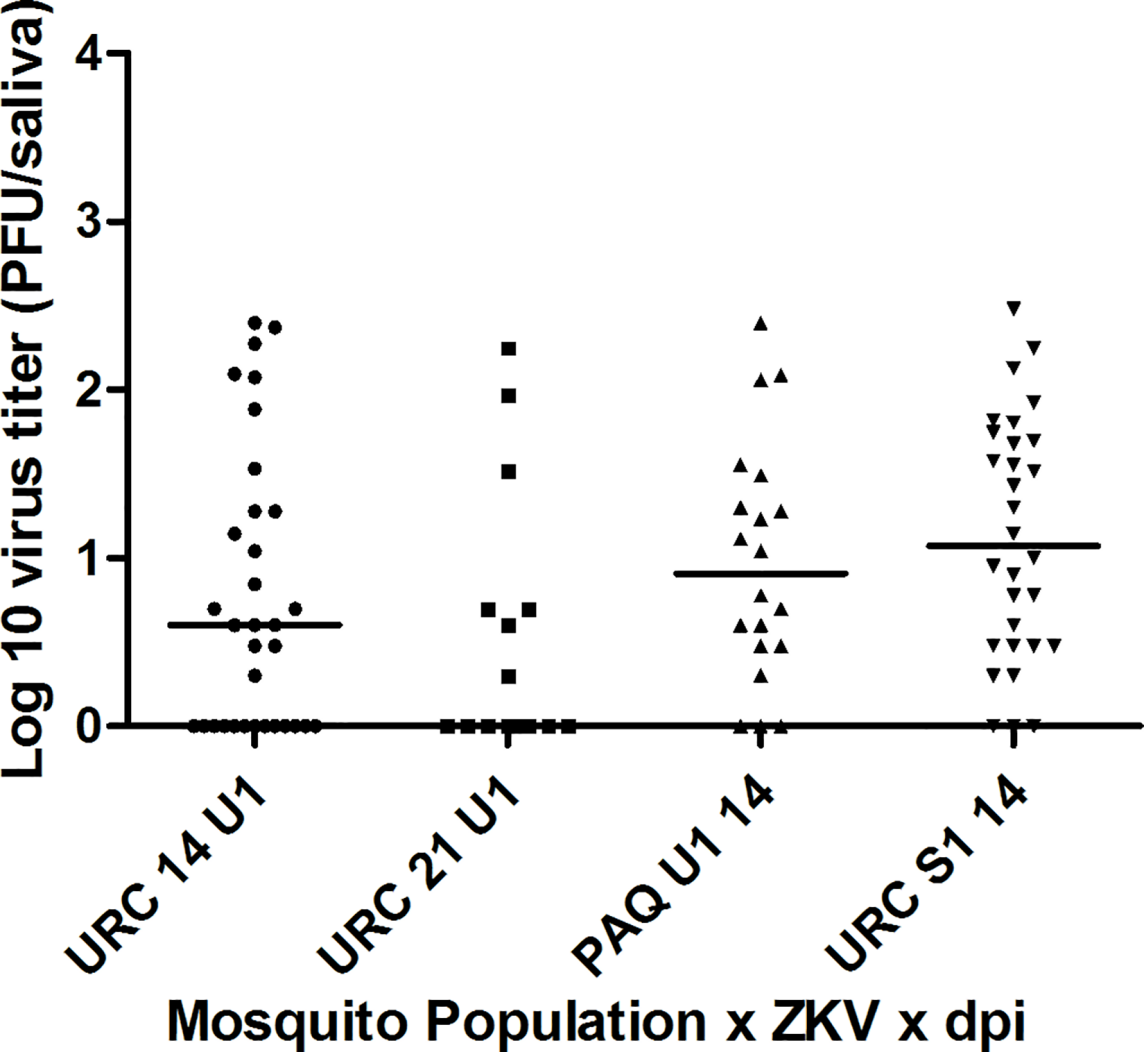
ZIKV load in saliva of *Ae*. *aegypti* from Rio de Janeiro, Brazil, 14 and 21 days after challenge with two locally circulating ZIKV isolates (Rio-U1 and Rio-S1) provided at a titer of 10^6^ PFU/mL. Virus was detected plaque forming unit (PFU) assays on Vero cells.

At 14 dpi, viral load in the head of *Ae*. *aegypti* from URC infected with ZIKV Rio-S1 (Fig 2) were significantly higher (median: 1.2 x 10^7^ RNA copies/mL; mean ± SE: 1.4 x 10^7^ ± 9.5 x 10^6^ RNA copies/mL) compared to ZIKV Rio U1 (median: 3.6 x 10^6^ RNA copies/mL mean ± SE: 6.3 x 10^6^ ± 7.8 x 10^6^ RNA copies/mL, Fig 2) (p = 0.0003). When challenged with the same ZIKV isolate (Rio-U1), viral load in heads at 14 dpi was significantly higher in *Ae*. *aegypti* from PAQ (median: 1.8 x 10^7^ RNA copies/mL, mean ± SE: 3.7 x 10^7^ ± 5.0 x 10^7^ RNA copies/mL, Fig 2) than URC (p = 0.0018). As expected, DIR was lower (DIR = 40%) in *Ae*. *aegypti* (URC) at 7 dpi, and no transmission was observed at this time point (Fig 1B–1D). TRs and TEs at 14 dpi were higher for PAQ compared to URC *Ae*. *aegypti* challenged with the same ZIKV isolate (Rio-U1) (Fig 1C and 1D), although viral load did not differ (p = 0.4203) between mosquito populations (Fig 3). Also, comparisons of viral loads in saliva of URC *Ae*. *aegypti* challenged with different ZIKV isolates did not show any difference (40.3 ± 64.5 PFU Rio-S1/saliva versus 34.2 ± 69.0 PFU Rio-U1/saliva; p = 0.3388) (Fig 3). No significant difference was apparent (p = 0.2212) in viral load in saliva between 14 and 21 dpi (Fig 3).

## Discussion

The Zika epidemics has affected nearly all American countries with ca. 445,000cumulative suspected cases, with 91,962 confirmed infections and 9 deaths due to ZIKV as of August 5, 2016 (http://ais.paho.org/phip/viz/ed_zika_cases.asp). South American countries had nearly 74% of the continental Zika suspected cases, with ca. 5% (165,932 suspected cases) from Brazil. The incidence rate in Brazil is 81.2/100,000 inhabitants Zika suspected cases, with 1,749 cases of microcephaly associated to ZIVK infection diagnosed by clinical, epidemiological and/or laboratory criteria as of May 2016 (http://www.paho.org/hq/index.php?option=com_content&view=article&id=11599&Itemid=41691). Rio de Janeiro is one of the highest risk areas in Brazil, with an incidence of 278.1/100,000 suspected Zika cases as of July 2016 (http://portalsaude.saude.gov.br/images/pdf/2016/julho/15/2016-boletim-epi-n28-dengue-chik-zika-se23.pdf).

To face such a severe health crisis, efficient and effective mosquito control strategies are essential. However, it depends on the definition of primary and/or potential local mosquito vectors. Other ZIKV transmission mechanisms besides *Ae*. *aegypti* have been observed. For instance, sexual ZIKV transmission between humans has been observed [35]. Natural ZIKV infections detected in several mosquito genera and even in horse flies would suggest that ZIKV could potentially infect a large range of mosquito species and even other hematophagous flies [31, 33, 36]. However, there is no evidence regarding the role of other mosquitoes or flies besides *Aedes* (*Stegomyia*) species in the ZIKV transmission in nature in the Americas. Indeed, there are no data whether other anthropophilic and domestic mosquitoes besides *Ae*. *albopictus*, and notably *Ae*. *aegypti* can transmit ZIKV.

In this work, we demonstrate for the first time, under laboratory conditions, that *Cx*. *quinquefasciatus* are not competent to transmit two ZIKV strains circulating in Brazil. Four tested populations were minimally infected with ZIKV and were unable to transmit this virus. In contrast, two *Ae*. *aegypti* populations were highly susceptible to ZIKV infection and dissemination, and competent to transmit the same virus strains. This is consistent with *Ae*. *aegypti* being more likely to sustain the current ZIKV outbreak in Rio de Janeiro and probably in other tropical American zones.

The Zika control program in Brazil, as well as in all epidemic American countries, consists essentially in intensifying and reinforcing the current strategies to control dengue for decades, which focuses in reducing *Ae*. *aegypti* density and longevity through eliminating or treating potential larval habitats and insecticide spraying (http://www.who.int/tdr/publications/documents/dengue-diagnosis.pdf). However, the traditional vector control strategies have usually failed to efficiently reduce dengue transmission and spread, even when properly adopted [38]. Several reasons have been identified to explain these failures, among which are insufficient community engagement and management and high insecticide resistance in the target species, the mosquito *Ae*. *aegypti* [39–41]. Intensifying *Ae*. *aegypti* control activities has also been unsuccessful in stemming the rapid spread of ZIKV [1]. Therefore, new technologies are urgently needed to adequately and better mitigate ZIKV transmission, likely requiring combinations of several approaches. For instance, it has been recently demonstrated that *Wolbachia*-infected *Ae*. *aegypti* from Brazil blocks ZIKV transmission [42]. In addition, local control programs should design specific control strategies against the potential vector *Ae*. *albopictus*, since it has been shown to transmit ZIKV in laboratory [25, 26, 37], with ZIKV detections in field-collected specimens [24, http://www.paho.org/hq/index.php?option=com_docman&task=doc_view&Itemid=270&gid=34243&lang=en].

The first determination of vector competence to ZIKV in American *Ae*. *aegypti* populations was conducted with a viral isolate from New Caledonia, as at the time of that evaluation, no local ZIKV strain were available. Nonetheless, the sequence of NS5 gene of ZIKV from New Caledonia displayed 99.4% identity with ZIKV from Brazil [26]. One Brazilian *Ae*. *aegypti* population, from Tubiacanga, Rio de Janeiro were challenged with the ZIKV New Caledonia. High susceptibility to infection and moderate dissemination rate, but with low transmission were found, suggesting unexpectedly low competence of local *Ae*. *aegypti* for ZIKV [26]. Our newly data with two *Ae*. *aegypti* populations from Rio de Janeiro (URC and PAC) orally challenged with two locally circulating ZIKV isolates (Rio-U1 and Rio-S1) revealed very high dissemination and moderate to high transmission. Similar results were found when testing the URC mosquito population with two ZIKV strains isolated in 2015 from other Brazilian cities [42]. These differences in vector competence may be explained by the concept that the outcome of transmission depends on the specific pairing of vector and virus genotypes [43]. Similar to other ZIKV strains isolated during the epidemic in Brazil, sequences of virus strains used in the present study clustered with Asian clade, including sequences from New World, Malaysia, Micronesia and Pacific. Thus, the New Caledonian [26] and Brazilian strains are genetically nearly identical. Phylogenetic and molecular clock analyses are consistent with a single introduction of ZIKV from the Pacific area into the Americas, probably more than 12 months before the detection of ZIKV in Brazil [21]. It is possible that some genome evolution not yet identified has rapidly shaped ZIKV to New World *Ae*. *aegypti* populations, highlighting the genetic specificity and potential for local adaptation between arboviruses and mosquito vectors previously described for dengue [44].

To evaluate the potential role of a mosquito species to transmit an arbovirus like ZIKV requires examination of multiple components governing vectorial capacity, of which vector competence is simply one. Ecological, epidemiological, environmental and climatic factors influence both vector competence and vectorial capacity. Thus, distinct geographical populations of a mosquito species can greatly diverge in their vector competence when exposed to different virus strains, since the outcome of infection depends on the specific combination of mosquito and virus genotypes [45, 46]. Thus, our demonstration that *Cx*. *quinquefasciatus* from Rio are not able to transmit ZIKV does not completely rule out the possibility that domestic *Culex* mosquitoes from other origins may exhibit different vector competence.

Nevertheless, to now at least, there is no evidence that the southern house mosquito *Cx*. *quinquefasciatus* is a potential ZIKV vector. Our study with four *Cx*. *quinquefasciatus* populations from Rio challenged with two recently isolated virus strains from the same location where mosquitoes were collected showed that this species is not competent to transmit ZIKV. Similar result was obtained when the closely related species *Cx*. *pipiens* from USA was challenged with a ZIKV isolated from Puerto Rico [47]. Moreover, besides being incompetent to transmit ZIKV in the laboratory, neither *Cx*. *quinquefasciatus* nor any other species of the Pipiens Assemblage has been found naturally infected in the American ZIKV transmission area [48, 49] or during the 2007 Zika outbreaks in the South Pacific island of Yap (Micronesia) [1, 16] and in Gabon [24] where thousands of *Cx*. *quinquefasciatus* have been screened.

Therefore, there is no reason to think that mosquito control efforts against *Cx*. *quinquefasciatus* to reduce Zika transmission, at least in Rio de Janeiro, Brazil. Mosquito measures to mitigate ZIKV transmission should remain focused on *Ae*. *aegypti*.
